# The Census of 1851, in Relation to Public and Private Lunatic Asylums

**Published:** 1851-01-01

**Authors:** Forbes Winslow, George Graham

**Affiliations:** Albermarle-street, Dec. 5, 1850


### THE CENSUS OF 1851, IN RELATION TO PUBLIC AND PRIVATE LUNATIC ASYLUMS.

*To the Editor of* The Lancet.

Sir,—As the subjoined correspondence between myself and the Registrar-General will be read with interest by a large class of professional gentlemen associated with public and private lunatic asylums, I offer no apology for transmitting it to you for publication in your journal.

It certainly would have been most unjustifiably inquisitorial, if the proprietors of private asylums for the insane had been compelled to make a return of the *names* of the patients placed in confidence under their care.

I remain, Sir, your obedient servant,
Forbes Winslow.

Dec. 9, 1850.

---

*To the Registrar-General.*

Sir,—I perceive, by the printed form of the census for 1851, issued, I believe, under your authority, that it will be necessary for each householder to make, agreeably to a prescribed tabular form, an accurate return of the Christian and surname, age, occupation in life, &c., of each person sleeping under his roof on a specified night. As I am much interested in behalf of a numerous and influential section of the medical profession—viz., those engaged in the care and treatment of the insane—may I be permitted to ask whether the resident proprietors or medical superintendents of private and public asylums will be required to make a return of the names of patients placed under their care, and actually resident with them at the period when it is proposed to take the census?

Your early attention to this matter will much oblige, Sir,
Your obedient servant,
Forbes Winslow, M.D.

Albemarle-street, Dec. 5, 1850.

---

Census Office, Craig's-court, Dec. 7, 1850.

Sir,—In reply to your letter of the 5th inst., I have to state that I should think that, rather than return the *names* of all patients in lunatic asylums, it may be sufficient if the initials of their names be recorded when the census of 1851 is taken, to which I should think there would be no objection.

I have the honour to be, Sir,
Your faithful servant,
George Graham,
Registrar-General.

Dr. Forbes Winslow, M.D.

